# RaPID2: a parallel scalable framework for identity-by-descent segment detection via parallel PBWT

**DOI:** 10.1093/bioadv/vbag078

**Published:** 2026-03-17

**Authors:** Kecong Tang, Ardalan Naseri, Degui Zhi, Shaojie Zhang

**Affiliations:** Department of Computer Science, University of Central Florida, Orlando, Florida, 32816, United States; McWilliams School of Biomedical Informatics, University of Texas Health Science Center at Houston, Houston, Texas, 77030, United States; McWilliams School of Biomedical Informatics, University of Texas Health Science Center at Houston, Houston, Texas, 77030, United States; Department of Computer Science, University of Central Florida, Orlando, Florida, 32816, United States

## Abstract

**Motivation:**

Identity-by-descent (IBD) detection plays a central role in genetic analysis, supporting applications such as genealogy, population history reconstruction, and disease gene mapping. RaPID, our previously developed IBD detection tool, demonstrated high detection power and accuracy. However, RaPID was not designed with high-performance computing (HPC) in mind and fails to scale efficiently to biobank-scale data. As large-scale datasets become increasingly common, parallel and distributed computing has become essential for maintaining performance at scale.

**Results:**

We present RaPID2, a redesigned and scalable IBD detection framework built for both HPC and memory-constrained environments. RaPID2 eliminates the disk I/O bottlenecks of its predecessor, enables memory-aware haplotype pair partitioning, and adopts a parallel and distributed architecture with fault-tolerant execution. It supports both fixed and dynamic window sizes and includes two runtime modes tailored for large-cluster deployment or smaller standalone machines. Our benchmarks show that RaPID2 achieves a 32-fold speedup over the original RaPID at a 2 cM threshold while maintaining statistically identical detection power and accuracy. RaPID2 provides a robust and efficient solution for IBD detection on modern genomic data scales.

**Availability and implementation:**

RaPID2 is implemented in C#. Its source code and software package are available at https://github.com/ucfcbb/RaPID2.

## 1 Introduction

The detection of identity-by-descent (IBD) segments plays a foundational role in population genetics, genealogy, and genome-wide association studies (Browning and Browning 2011). IBD segments are genomic regions shared between two or more individuals that are inherited from a common ancestor. Identifying such regions enables the study of fine-scale population structure (Han *et al.* 2017), relatedness (Henn *et al.* 2012), and demographic history (Palamara *et al.* 2012). As biobank-scale datasets (Sudlow *et al.* 2015, Stoeklé *et al.* 2016, All of Us Research Program Investigators 2019) grow to encompass millions of haplotypes and tens of millions of variants, existing IBD detection methods increasingly face computational and scalability bottlenecks (Dean and Ghemawat 2008, Zaharia *et al.* 2010). While prior approaches have scaled IBD inference to large cohorts, trade-offs remain between detection resolution, windowing flexibility, and computational efficiency. Among these approaches, RaPID (Naseri *et al.* 2019) is a positional Burrows–Wheeler transform (PBWT)-based (Durbin 2014) method by finding exact matches within low-resolution random projections of the original sequences. This allows for tolerance of mismatches (errors or mutations) and controls false positives by considering only those matches that consistently appear across several projections. RaPID demonstrated high detection power with only minor accuracy trade-off (Tang *et al.* 2022), but its reliance on intermediate IO files incurred substantial disk overhead, ultimately limiting its runtime performance.

RaPID2 is a redesigned and re-engineered implementation of the RaPID framework, aimed at achieving high-throughput IBD detection across modern computational architectures. Building on the core methods of RaPID, RaPID2 integrates HP-PBWT (Tang *et al.* 2025), a high-performance redesign of the original PBWT, which departs significantly from the original formulation to support multi-threaded, memory-aware execution over large cohorts. This upgrade, along with algorithmic changes in match detection, merging, and dataflow organization, results in significantly improved runtime performance while preserving the accuracy and flexibility of the original method.

The pipeline in RaPID2 is structured into modular stages: (i) generation of randomized downsampling projections Random Projection Layer to enhance IBD sensitivity, (ii) high-performance parallel matching via HP-PBWT Layer, (iii) cache-optimized region-aware long match detection, (iv) lock-free match accumulation using parallel-friendly sorting Lock-Free Match Accumulation and Merging, and (v) a linear overlap merge Overlap Merging in the validation and merge stage. Each of these stages is designed with computational resource constraints in mind and can be tailored to diverse hardware environments.

To support deployment across heterogeneous compute systems, RaPID2 offers two configurable execution strategies: (i) a Memory-Retentive HPC mode, which retains all intermediate data structures in memory for maximum throughput; and (ii) a Memory-Stable mode, which processes each genomic subregion independently to minimize memory usage. These two modes share the same algorithmic logic and produce identical outputs, differing only in system-level performance strategies. The Memory-Stable mode is designed for execution on resource-constrained machines, minimizing memory usage through repeated computation. In contrast, the Memory-Retentive mode leverages large-memory high-performance systems to maximize throughput by caching intermediate data structures. This dual-mode flexibility enables RaPID2 to scale across a wide spectrum of computing environments, from small personal workstations to large-scale HPC clusters. The design reflects classical trade-offs in parallel computing, balancing memory-bounded speedup (Sun and Ni 1993) with adaptive memory utilization strategies similar to DynIMS (Xuan *et al.* 2016).

Benchmarking against both RaPID and hap-IBD (Zhou *et al.* 2020) demonstrates that RaPID2 achieves up to 32-fold runtime improvement over RaPID on UK Biobank chromosome 20 at a 2 cM threshold, while remaining competitive with hap-IBD in both speed and power. Moreover, our cost modeling indicates that RaPID2 offers substantial savings in cloud environments, reducing estimated EC2 compute costs by over 70% in typical configurations and over 95% when accounting for IO acceleration via in-memory computation.

In summary, RaPID2 delivers a practical, high-performance solution for scalable IBD inference. It combines methodological rigor with systems-aware implementation, enabling researchers to perform accurate long-range relatedness detection across large-scale genomic cohorts with orders-of-magnitude improvement in efficiency.

## 2 Methods

The design of RaPID2 addresses several key challenges: enabling PBWT-based match detection while tolerating mismatches, substantially improving PBWT execution speed, maintaining flexibility across different computational resource scales, and minimizing redundant computation as much as possible. RaPID2 is designed as a modular pipeline for rapid and memory-aware detection of identical-by-descent (IBD) segments in large genomic datasets. It builds upon the core logic of its predecessor, RaPID, but introduces substantial algorithmic and architectural improvements. The method is composed of four primary stages: (i) randomized haplotype downsampling to facilitate approximate detection of IBD segments in the presence of genotyping error, (ii) haplotype-based parallel PBWT execution incorporating divergence profiling, (iii) partitioned match detection and accumulation, and (iv) computationally efficient segment validation and merging. Each of these stages is parallelizable and has been engineered to support memory-bounded execution across diverse hardware configurations.

RaPID2 implements two execution modes to accommodate varying computational environments. The *Memory-Retentive HPC Mode* retains intermediate PBWT structures in memory and is optimized for high-throughput, memory-rich systems. The *Memory-Stable Mode*, by contrast, decomposes the computation into smaller, memory-efficient sub-jobs that can be executed either sequentially or distributed across independent machines. This design reduces peak memory usage while incurring additional computational effort, a deliberate trade-off of space for time (Pagter and Rauhe 1998). Both modes are compatible with distributed execution and produce identical outputs under the same parameters. This flexibility reflects a fundamental principle in computing and system design: that time and space, while independently constrained, can be strategically exchanged to meet environmental limits (Ding *et al.* 2024, Vaithianathan *et al.* 2025).

### 2.1 Random projection layer

The first stage of RaPID2 involves reducing the input genotype panel into multiple lower-resolution representations, each generated through a process of randomized downsampling. This projection step is designed to retain long-range haplotype information while increasing resilience to genotyping errors and local inconsistencies. By applying this transformation multiple times with different random seeds, RaPID2 achieves high sensitivity in detecting identical-by-descent (IBD) segments even under imperfect or sparse input conditions.

RaPID2 supports both fixed-size and genetic-length-based dynamic windows for projection. In the fixed-window mode, the input panel is divided into uniform, contiguous blocks of equal site count, typically selected to maintain computational balance and memory alignment. This approach aligns with the random windowing scheme used in RaPID.

In contrast, the dynamic-window mode divides the genome into intervals of approximately constant genetic distance, such as 0.05 cM, using a genetic map to adaptively assign boundaries. This allows better alignment with recombination rates and more uniform biological interpretation across the genome. To ensure reliable matching, a minimum site threshold is enforced during window generation; if a dynamic interval contains too few sites, adjacent regions are merged until the constraint is met. RaPID2 also supports *hybrid configurations*, where dynamic genetic-length criteria are combined with fixed minimum site requirements. This balances biological sensitivity with computational robustness, producing intervals that are both interpretable and data-dense. Fixed, dynamic, and hybrid projection modes can each be repeated independently using different random seeds, producing a diverse set of downsampled haplotype panels for downstream matching.

### 2.2 HP-PBWT layer

One of the main challenges in IBD detection is the increasing number of haplotypes in the datasets. HP-PBWT is well suited to address this challenge, enabling the processing of up to billions of haplotypes (Tang *et al.* 2025). RaPID2 integrates an enhanced haplotype-matching engine built on HP-PBWT (Haplotype-based Parallel PBWT), a cache-aware, multi-threaded, re-implementation of the classical PBWT framework. While the classical PBWT performs haplotype match scanning in O(MN) time for *M* haplotypes across *N* variant sites, HP-PBWT introduces a multi-threaded design that partitions query workloads across *T* concurrent processing units. This parallelization reduces the effective runtime to approximately O(MN/T). This parallelization follows long-established principles in partitioned computing and workload balancing (Devine *et al.* 2006, Bokhari 1978) and is specifically tailored for high-throughput genomic data. HP-PBWT preserves the core prefix sorting and divergence tracking mechanisms of PBWT, but reconfigures their execution order and memory layout to enable efficient, thread-safe traversal and update under parallel execution.

### 2.3 Prefix array

At each site indexed by *k*, the algorithm maintains a prefix array $P_{k}$, which encodes the co-lexicographic ordering of haplotypes based on their reversed prefixes up to the current site. Formally, let $\left\{x_{i}\right\}$, where i=1 to *M*, denote *M* haplotype sequences over *N* sites. At site *k*, the **prefix array**  $P_{k}$ is defined as the permutation of sequence indices obtained by sorting the sequences according to their reversed prefixes $x_{i}[0,k)$. Thus, $P_{k}[i]$ gives the index of the *i*th sequence in the co-lexicographic ordering at position *k*. This ordering is central to the PBWT’s ability to efficiently detect shared segments. In HP-PBWT, the update of $P_{k}$ is parallelized using a three-stage prefix-sum procedure comprising (i) local scan, (ii) offset propagation, and (iii) adjustment. This structure mirrors the classic tree-based scan model (Blelloch 1989) and is conceptually analogous to hardware-level parallel-prefix adders such as the Kogge–Stone architecture (Kogge and Stone 1973). Together, these stages enable efficient and thread-safe updating of the prefix array across sites. The per-site runtime is reduced from the original O(M) complexity in the sequential PBWT to approximately O(M/T+T), where *M* is the number of haplotypes and *T* is the number of threads. This design facilitates scalable performance on modern multi-core systems.

### 2.4 Divergence array

The divergence array $D_{k}$ indicates how long two adjacent haplotypes in the prefix ordering $P_{K}$ match to each other. Formally, for adjacent sequences in the prefix ordering, the **divergence array**  $D_{k}[i]$ records the starting position of the longest common suffix of $x_{P_{k}[i-1]}$ and $x_{P_{k}[i]}$ ending at site k−1. Specifically, $D_{k}[i]$ is the smallest position *j* such that $x_{P_{k}[i-1]}[j,k)=x_{P_{k}[i]}[j,k)$. This information encodes the length of exact shared haplotype matches and is essential for identifying the boundaries of long identical-by-descent (IBD) segments. In HP-PBWT, divergence updates are parallelized by dividing haplotypes into blocks assigned to individual threads. Within each block, the divergence values are updated locally in parallel, achieving a per-site runtime of approximately O(M/T), where *M* is the number of haplotypes and *T* is the number of threads.

To maintain full parallelism, each block must operate independently without waiting for divergence values from preceding blocks, otherwise, the execution would become inherently sequential. However, computing divergence requires information from previously sorted haplotypes. To address this, HP-PBWT computes per-block divergence seeds in parallel. These compact summaries, typically block-level minima, capture key divergence characteristics locally and are propagated downstream to initialize divergence computation in the next block. This mechanism avoids costly O(M) backward scans and draws inspiration from communication-avoiding scan techniques such as decoupled look-back strategies (Merrill and Garland 2016). It enables correct divergence tracking across blocks while preserving parallel scalability, improving cache locality, reducing synchronization overhead, and promoting balanced thread workloads.

### 2.5 Long match detection

As PBWT progresses along sites, long matches are reported for haplotype pairs when they become separated in the prefix array, meaning they no longer match. Given a minimum match length *L*, a **long match** between two sequences is defined as a shared contiguous segment of length at least *L* ending at site *k*. In PBWT, such matches correspond to the contiguous intervals in the prefix array ordering $P_{k}$ in which, for all positions *m* within the interval where adjacent sequences satisfy $D_{k}[m]\leq k-L$. PBWT reports all such long matches by scanning these intervals and outputting matches when adjacent sequences no longer match at the next site. In HP-PBWT, the long match detection stage identifies all pairwise exact matches among haplotypes in the order defined by the prefix array. Because the output consists of raw segment coordinates with no additional post-processing, this per-haplotype reporting strategy is computationally efficient and well-aligned with CPU execution. Each thread independently scans its assigned subset of haplotypes, resulting in a balanced workload and minimal synchronization overhead.

In contrast, RaPID2 extends beyond raw match reporting and must support additional downstream computations, including segment verification and merging to generate finalized IBD calls. These subsequent stages introduce specific constraints on data flow and memory locality, which in turn influence the optimal strategy for long match detection.

To address these requirements, RaPID2 adopts a region-aware design that prioritizes CPU cache locality and dynamic workload partitioning. Specifically, the long match detection stage is restructured to first identify regions of interest (ROIs), genomic intervals likely to contain dense matching activity, before performing pairwise comparisons. While the original PBWT implicitly employed a similar strategy by limiting its inner loop to contiguous divergence-preserving ranges, it did not formalize or expose these regions as units of computation. RaPID2 builds upon this concept by explicitly detecting ROIs in parallel and assigning each to a dedicated thread for localized, cache-friendly execution, which is critical to preserving performance as core counts increase (Herlihy and Liu 2013).

To align ROI detection with biologically meaningful distance thresholds, RaPID2 defines each region based on genetic distance rather than physical site count. At each genomic position *s*, a lookup is performed against the genetic map to determine the number of preceding sites that span the desired cut-off (e.g. 2 cM or 5 cM). This is accomplished via a lightweight linear scan in reverse from site *s* to identify the earliest site $s^{\prime }$ such that the cumulative genetic distance from $s^{\prime }$ to *s* meets or exceeds the threshold. The resulting site window length (e.g. 20 sites for a 2 cM span) is then used to define the minimal segment length required to trigger an ROI.

Next, the divergence array $D_{k}$ is scanned in the order defined by the prefix array $P_{k}$, which preserves the local sorting of haplotypes. For implementation convenience, divergence values are accessed using haplotype identifiers mapped through the prefix array. For each haplotype at site *s*, if the divergence length $D_{k}[P_{k}[i]]$ exceeds the derived site-length threshold, the position is flagged as the start of an ROI. The scan continues until divergence falls below the threshold, marking the end of the ROI. This detection process is executed in parallel across blocks, yielding an O(M/T) runtime, where *M* is the number of haplotypes and *T* is the number of threads. After scanning, the candidate ROIs from each thread are merged in O(T) time to form the final set of disjoint regions for long match verification.

Overall, while HP-PBWT’s per-haplotype parallelism remains a general-purpose solution, RaPID2’s ROI-parallel strategy is specifically optimized for IBD inference, where memory reuse, execution granularity, and data-aware scheduling are critical to scalability.

### 2.6 Lock-free match accumulation and merging

Following long match detection, RaPID2 enters a match accumulation and verification stage in which exact-match segments are collected, grouped, and transformed into valid IBD segments. Each detected match is recorded as a tuple (A,B,r,S,E), denoting a pair of haplotypes (A,B) that match from site *S* to *E* in the context of a specific random projection *r*.

To enable downstream verification and merging, all matches must be organized according to their originating haplotype pair and projection index. Specifically, the algorithm requires that all (S,E) segments for a given (A,B) pair under projection *r* be ordered by their start coordinates to enable efficient interval merging. This ordering is required for correctness and efficiency. Although it can be achieved through sorting, hashing, or other indexing structures, the need for key-based organization is inherent to the computation itself rather than to a particular implementation choice. While data structures such as nested dictionaries or concurrent hash tables may appear lightweight under conventional workloads, they are ill-suited for high performance computing (HPC) environments. These approaches typically involve disorganized insertions followed by internal key-based sorting, executed under lock contention or cache-inefficient memory access. In practice, operations such as *put* and *get* can degrade dramatically under high concurrency, as lock contention leads to thread queuing and unpredictable memory pressure (Herlihy and Shavit 2008). This overhead can obscure actual computation cost and cause instability at scale.

RaPID2 adopts a more cache-aware and deterministic strategy using a two-layered sorting framework. First, a coarse-grained *bucket sort* groups all match records by their (A,B) haplotype pair using the integer *A* as a primary key. This ensures that all records associated with the same haplotype pair are placed in the same bucket. To promote even workload distribution across processing threads, the number of buckets is set to a multiple (e.g. 2× or 3×) of the available core count.

After the initial bucketing phase, each bucket is processed in parallel using an internal O(n log ⁡n) sort to order its match intervals by start site *S*. This guarantees that segments associated with each (A,B,r) key appear in proper order, ready for verification and merging. RaPID2 leverages the system’s built-in sorting routines, typically introsort-based implementations (Musser 1997), which are highly optimized for modern CPU architectures and benefit from years of compiler-level tuning. In practice, the effective runtime for this stage scales approximately as O((n log ⁡n)/T), where *n* is the total number of match intervals and *T* is the number of cores assigned to sorting.

### 2.7 Match validation

After match collection, RaPID2 enters the validation stage. The core idea is that true IBD segments should appear consistently across multiple random projections, while spurious matches caused by noise or local alignment anomalies such as phasing errors or genotyping error are likely to be isolated.

For each segment between a haplotype pair found in one projection, RaPID2 checks whether sufficiently overlapping segments are found in other projections. If the same pair shares a segment of similar position and length in at least s projections (typically s=2), the segment is considered valid and retained. Otherwise, it is discarded as likely spurious. This threshold is user-adjustable, offering a trade-off between sensitivity and specificity: higher thresholds reduce false positives but may miss borderline segments; lower thresholds preserve power but may admit some short or weakly supported segments.

The validation logic is similar in spirit to RaPID, which modeled projection consistency using binomial probabilities. RaPID2 adopts the same principle, but performs the comparison in-memory across pre-indexed projection collections, enabling faster filtering even at biobank scale.

### 2.8 Overlap merging

Following match validation, RaPID2 performs a merging step to consolidate overlapping segments into final IBD calls. Each haplotype pair (A,B) is associated with multiple sorted lists of candidate segments (S,E), one per random projection. These segments are internally consistent and non-overlapping within each list.

Algorithm 1 Linear Overlap Merge

**Require:** Two sorted lists of non-overlapping ranges: setA, setB

**Ensure:** A merged sorted list of non-overlapping ranges

 ptrA  ←0;  ptrB  ←0

 temOld  ←[−1,−1];  tem  ←[−1,−1]

 merged  ← empty list

** while**  ptrA<setA.length  **and**  ptrB<setB.length  **do** 

**  if**  setA[ptrA].Start<setB[ptrB].Start  **then**  ▹ Pick a group

   tem  ←setA[ptrA]

   ptrA  ←ptrA  +1

**  else** 

   tem  ←setB[ptrB]

   ptrB  ←ptrB  +1

**  end if** 

**  while**  tem≠temOld  **do**  ▹ Merge

   temOld  ←tem

**   while**  Overlap(tem, setA[ptrA]) **do**  ▹ First group

    tem  ←  MergeRange(tem, setA[ptrA])

    ptrA  ←ptrA  +1

**   end while** 

**   while**  Overlap(tem, setB[ptrB]) **do**  ▹ Second group

    tem  ←  MergeRange(tem, setB[ptrB])

    ptrB  ←ptrB  +1

**   end while** 

**  end while** 

  merged.Add(tem) ▹ Collect merged

** end while** 

** while**  ptrA<setA.length  **do**  ▹ Collect remaining

  merged.Add(setA[ptrA])

  ptrA  ←ptrA  +1

** end while** 

** while**  ptrB<setB.length  **do** 

  merged.Add(setB[ptrB])

  ptrB  ←ptrB  +1

** end while** 

**  return** merged

Since match candidates are already sorted in the previous stage and stored in memory, RaPID2 no longer loads match candidates from hard drive and then employs an interval tree sorting as RaPID, which incurs a merging complexity of O(n log ⁡n). RaPID2 exploits the sorted nature of the inputs to perform a *Linear Overlap Merge*: a sweep-based procedure that iteratively absorbs overlapping segments across lists while preserving order. This method ensures that: all overlapping or adjacent segments across projections are merged with linear overhead. The final merged output remains sorted and non-redundant. Computational complexity is reduced to O(n), where *n* is the total number of input segments across projections.

Although the conceptual explanation and Algorithm 1 illustrate the core logic for merging two lists, the full implementation is generalized naturally to *n* input lists, typically ten in this study. This generalization preserves the linear-time nature of the merge while introducing modest overhead for heap-based candidate selection or index-based scanning. The merged segments are then streamed directly to disk using a buffered writer, avoiding global locking or coordination bottlenecks. This in-place, memory-efficient output strategy distinguishes RaPID2 from earlier methods such as RaPID, which relied on disk-heavy intermediate storage and post-processing via interval-based structures.

### 2.9 Memory-aware partitioned execution

**Figure 1:**
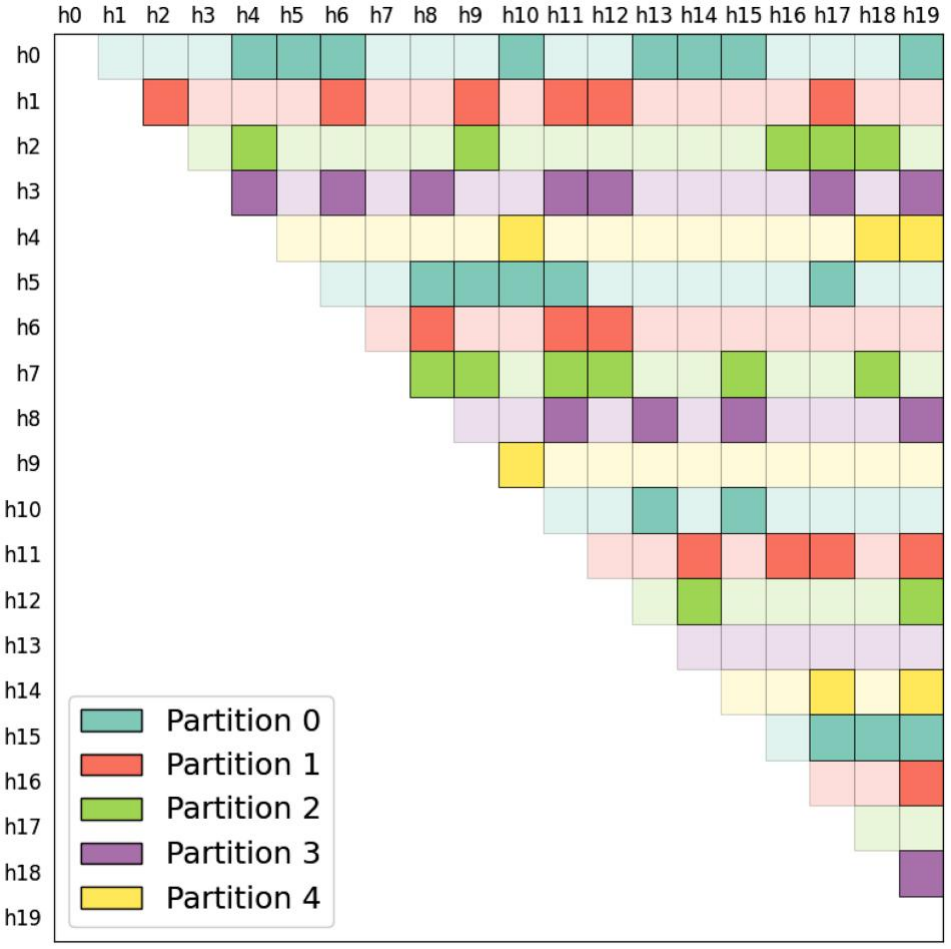
Partitioning strategy for scalable processing in RaPID2. RaPID2 partitions the triangular haplotype pair space based on the condition A%P=p, where *A* and *B* are haplotype IDs, and A<B, *P* is the total number of partitions, and *p* is the current partition index. Different partitions could be executed sequentially in a same machine or executed simultaneously in a distributed environment. For a given partition, only the pairs satisfying this condition A%P=p are processed. Each colored cell corresponds to a pair assigned to a specific partition. Faded cells represent haplotype pairs with no detected IBD segments, for which no memory is allocated. The size of each partition is determined by the chosen number of partitions; increasing this number yields smaller partitions and lower memory usage, while fewer partitions produce larger workloads that make fuller use of available memory.

To support memory-efficient execution without compromising the all-vs.-all nature of IBD detection, RaPID2 adopts a partitioning strategy that operates on haplotype pairs (Gusev *et al.* 2009) rather than subpanels. All pairs are normalized such that A<B to ensure consistent key assignment, and each pair is deterministically assigned to a partition using the condition A%P=p, where *P* is the total number of partitions and *p* is the current partition index. This guarantees a complete and non-redundant decomposition of the upper-triangular pair space. During execution, only those pairs that belong to the current partition are processed, reducing peak memory load. Since match detection is performed dynamically, not all pairs yield results; unmatched pairs are effectively skipped. Figure 1 illustrates this scheme, with color indicating partition assignment and faded cells representing unmatched pairs.

This partitioning framework ensures that the computational and memory load remains bounded within each partition, making the approach scalable to datasets with millions of haplotypes. The same codebase can be executed on a single high-memory server or distributed across a compute cluster with commodity hardware. No global memory sharing is required between partitions, and the pipeline is inherently parallelizable.

Importantly, even users with access to only a single low-memory machine can complete the full analysis by increasing the number of partitions. Each partition requires only a fraction of the total memory, and partitions can be processed sequentially without global state. This makes RaPID2 usable in constrained environments, such as desktops or cloud instances with limited RAM.

In cases where a specific partition exhibits unusually high memory usage or extended runtime, due to, for example, elevated haplotype density or overlapping IBD structure, the user has two options: (i) execute that partition independently on a larger machine, or (ii) subdivide the partition further. Both strategies preserve correctness while enabling adaptive recovery from performance bottlenecks.

Additionally, when deploying RaPID2 on shared or high-performance machines, users may wish to maximize hardware utilization by tailoring the granularity of partition execution. For panels with low per-partition memory and CPU usage, it is advantageous to run multiple partition instances concurrently on the same node. Conversely, for panels that exhibit high memory locality or compute intensity, a single larger partition may yield better throughput and reduced IO contention. This flexibility allows the tool to adapt to both panel characteristics and system architecture, improving efficiency in large-scale production workflows.

Match detection within each partition is performed using RaPID2’s cache-aware long match detection engine, a redesigned and parallelized version of the PBWT-based core. This engine traverses the panel to identify long, shared haplotype segments while preserving prefix consistency and divergence tracking across sites. By incorporating optimizations such as region-aware memory access, multi-threaded prefix array updates, and divergence seeding, the algorithm achieves substantial performance improvements over previous sequential methods (see Algorithm 2 for details).

Algorithm 2 Report Long Matches Within Partition

**Require:** Total number of random projections *nRDP*, current partition *p*, total number of partitions *P*

**Ensure:** Match segments stored in holder[*A*][*B*][$RDP_{i}$]

** for**  $RDP_{i}\leftarrow 0$  **to** *nRDP* **do** 

**  for all**  s∈selectedSites[$RDP_{i}$] **do** 

   siteIndex←selectedSiteIndexes[$RDP_{i}$][*s]*

   ComputePrefixArray(*siteIndex)*

   ComputeDivergenceArray(*siteIndex)*

   oneSite←panel[siteIndex+1*]*

**   for**  a←0  **to**  nHap−2  **do** 

**    for**  b←a+1  **to**  nHap−1  **do** 

**     if**  mLens[pArr[b]]<Threshold  **then** 

**      break** 

**     end if** 

     length←min⁡(length,mLens[pArr[b]])

**     if**  pArr[a]<pArr[b]  **then** 

      A←pArr[a]; B←pArr[b]

**     else** 

      A←pArr[b]; B←pArr[a]

**     end if** 

**     if**  A%P≠p  **then** 

**      continue** 

**     end if** 

**     if**  oneSite[pArr[a]]≠oneSite[pArr[b]]  **then** 

      holder[*A*][*B*][$RDP_{i}$] ←segment

**     end if** 

**    end for** 

**   end for** 

**  end for** 

**end for** 

### 2.10 High-performance and memory-awareness

**Figure 2:**
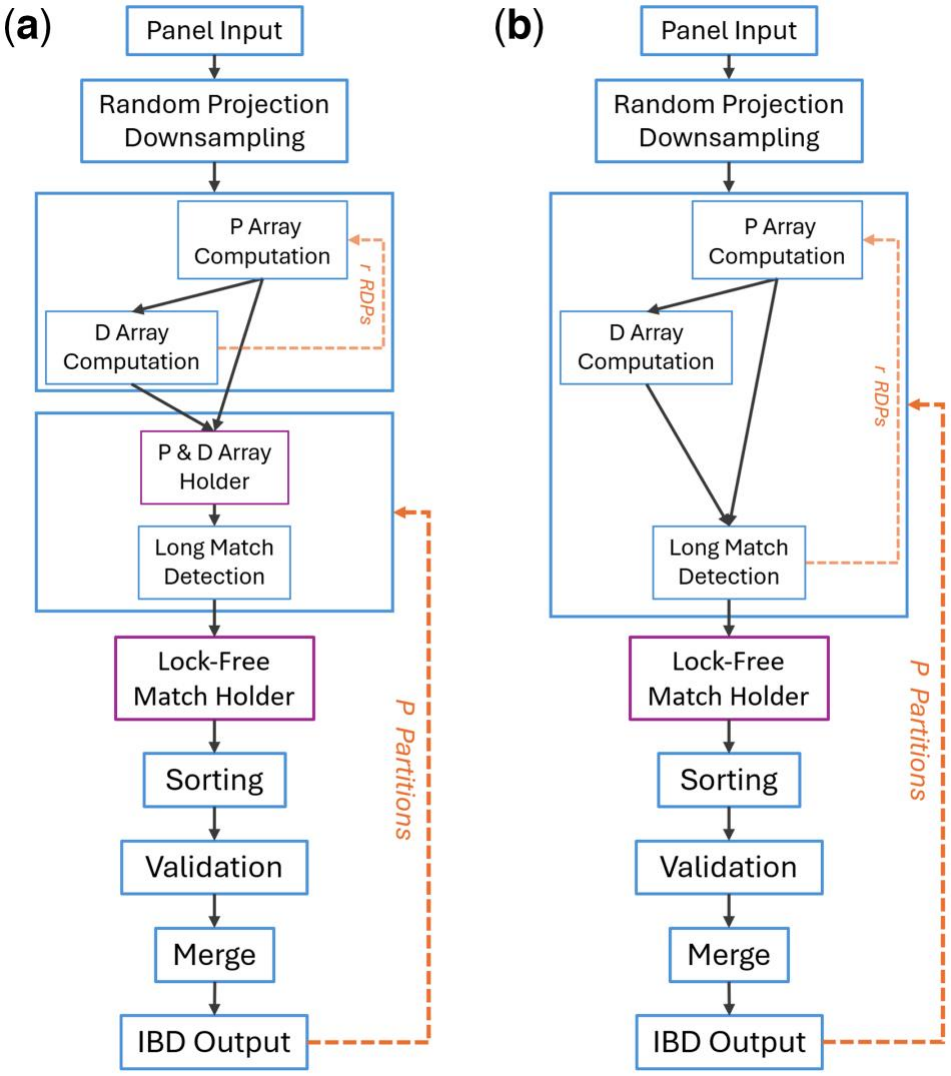
Flowchart comparison of the two execution modes in RaPID2. The term “*r* RDPs” refers to *r* times of Random Projections. (a) The Memory-Retentive (HPC) mode stores all prefix (P) and divergence (D) arrays after initial computation, allowing downstream stages to reuse these data structures across partitions. This improves throughput but requires sufficient memory. (b) The Memory-Stable mode recalculates the P and D arrays for each random projection and each partition, reducing peak memory usage at the cost of redundant computation. In both modes, detected matches from the long match stage are collected using a lock-free holder, then sorted, verified, merged, and written to output. The dashed arrows indicate repeated steps across projections or partitions. The main difference between the two modes is that in HPC mode the P and D arrays are computed once and reused directly from memory, whereas in memory-stable mode, they are repeatedly recalculated to minimize memory usage.

RaPID2 introduces two memory-aware execution modes to accommodate a wide range of computing environments. Both configurations rely on partitioning the dataset into manageable segments but differ in how intermediate data are held and reused across partitions. Figure 2 and Table 1 provide overall workflow and time-space trade-off between the Memory-Retentive mode and the Memory-Stable mode. The Memory-Retentive mode stores computed prefix and divergence arrays, resulting a memory complexity of O(M·n·r+c) while avoiding redundant recomputation. In contract, the Memory-Stable mode recalculates prefix and divergence arrays when needed, reducing memory usage to O(M+c). Here, *M*, *n*, and *c* denotes the number of haplotypes, sites, and number of match respectively.

**Table 1:** Number of computational passes per pipeline stage under two execution modes of RaPID2.^a^

Pipeline stage	HPC mode	Stable mode
Load panel	1	*p*
P array computation	*r*	r·p
D array computation	*r*	r·p
Long match detection	r·p	r·p
Validation and merge	*p*	*p*

a Here, *p*, *r*, *M*, and *n* denotes the number of partitions, random projections, haplotypes, and sites respectively.

### 2.11 Memory-retentive mode

In the Memory-Retentive (HPC) mode, the full reference panel and all precomputed random projections as well as computed P and D arrays are loaded into memory once and retained across the entire execution span. The pipeline then iterates over all partitions, performing Long Match (L), Verification (V), and Overlap Merge (O) steps. This configuration is designed for high-memory systems where memory management is stable and predictable. By avoiding repeated data loading and reinitialization, this mode achieves maximal throughput and is ideal for tightly coupled HPC clusters or high-performance workstations.

However, this mode assumes the underlying system can consistently reclaim unused memory. In environments where memory is not properly released, due to garbage collection stalls or memory leaks in long-lived processes, the system may eventually experience memory exhaustion. In our empirical observations, some distributed or containerized servers failed to complete tasks under this mode despite having sufficient nominal memory, suggesting that memory fragmentation and OS-level allocation policies can pose practical limits. Recent studies characterize such fragmentation as a hidden barrier: even when free memory exists, it may be too fragmented to satisfy large allocations, causing applications to fail or degrade sharply under memory pressure (Mansi and Swift 2024).

### 2.12 Memory-stable mode

The Memory-stable mode prioritizes robustness and cross-platform consistency. For each partition, the reference panel is freshly loaded, then random projections are generated per partition. Each execution loop performs Prefix Array (P), Divergence Array (D), and Long Match (L) steps across all random projections, followed by the Verification (V) and Overlap Merge (O) phases. After completing a partition, the program restarts, releasing all memory before continuing to the next partition.

Although this approach introduces some overhead due to repeated file IO and process startup, it significantly reduces the risk of memory-related execution failures. To mitigate this cost, RaPID2 incorporates a custom-designed and highly optimized loading pipeline developed by our team, capable of converting a 30 GB VCF file to in-memory binary format in under 2 minutes on a typical server node. This mode is particularly advantageous for systems with aggressive memory reclamation issues or for users operating in shared memory environments. Despite being modestly slower, it ensures stability across a wide range of platforms and is the default recommendation for long-term, large-scale deployments.

Furthermore, partitioning can be adapted based on input data characteristics. Datasets with high IBD density (e.g. sequencing panels, short cM cut-offs) tend to produce more segments, requiring finer partitioning. Conversely, sparse panels or higher cM thresholds may allow larger partitions. Users can also monitor CPU utilization across partition runs to identify optimal load distribution, some hardware may parallelize better with larger partitions, others with smaller ones.

## 3 Results

### 3.1 Power and accuracy validation

Before evaluating runtime and memory usage on UK Biobank data, we first verify that RaPID and RaPID2 yield identical power and accuracy, since the main purpose of this work is to introduce the design and implementation of a new high-performance parallel algorithm for IBD detection. We validated this equivalence by repeating the same experimental protocols used in the open-source IBD detection benchmarking project (Tang *et al.* 2022), which provides standardized comparisons for biobank-scale cohorts. The validation used the simulated European (EUR) dataset from out-of-Africa population model (Gutenkunst *et al.* 2009) generated with msprime under a 0.1% genotyping error rate, as defined in the benchmarking study.

**Figure 3:**
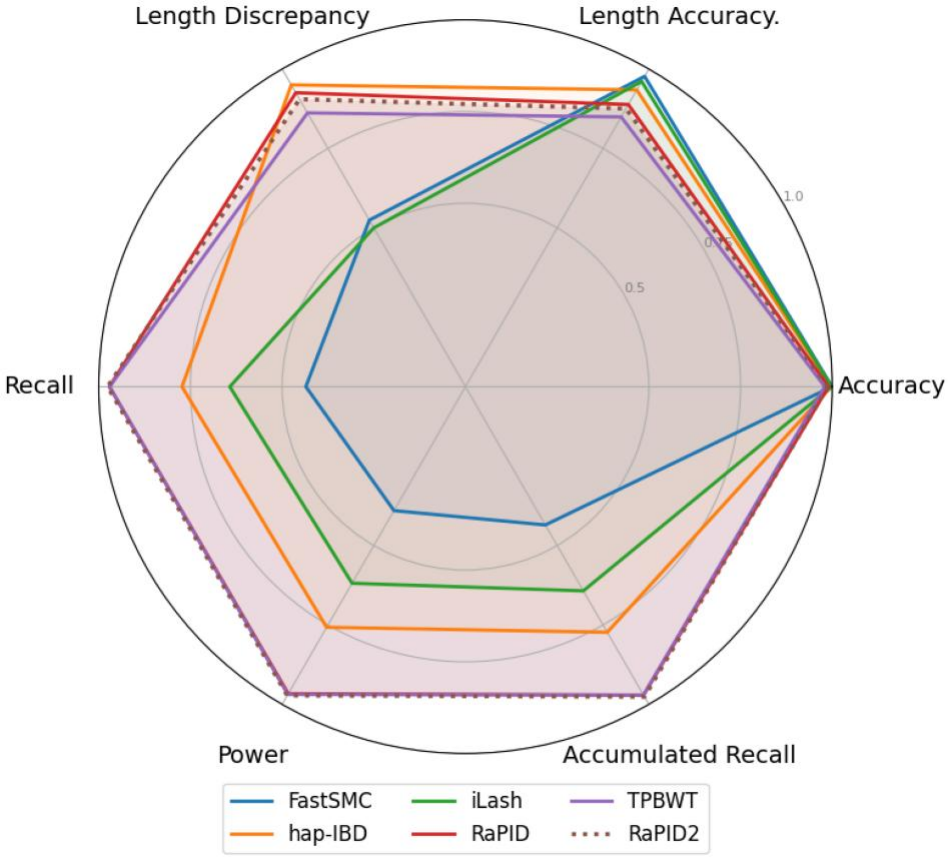
Power and accuracy results at a 2 cM length threshold from the IBD segment detection tool benchmarking project, evaluated on msprime OOA model EUR array data (Kelleher *et al*. 2016) with a simulated genotyping error rate of 0.1%. The *Length Discrepancy* is normalized from genetic distance and quantifies the agreement between detected and true IBD segment lengths; all other values are reported as percentages. Raw data for this figure is provided in Table 1, available as supplementary data at *Bioinformatics Advances* online. The parameters and command lines used for each tool are provided in Table 3, available as supplementary data at *Bioinformatics Advances* online.

**Figure 4:**
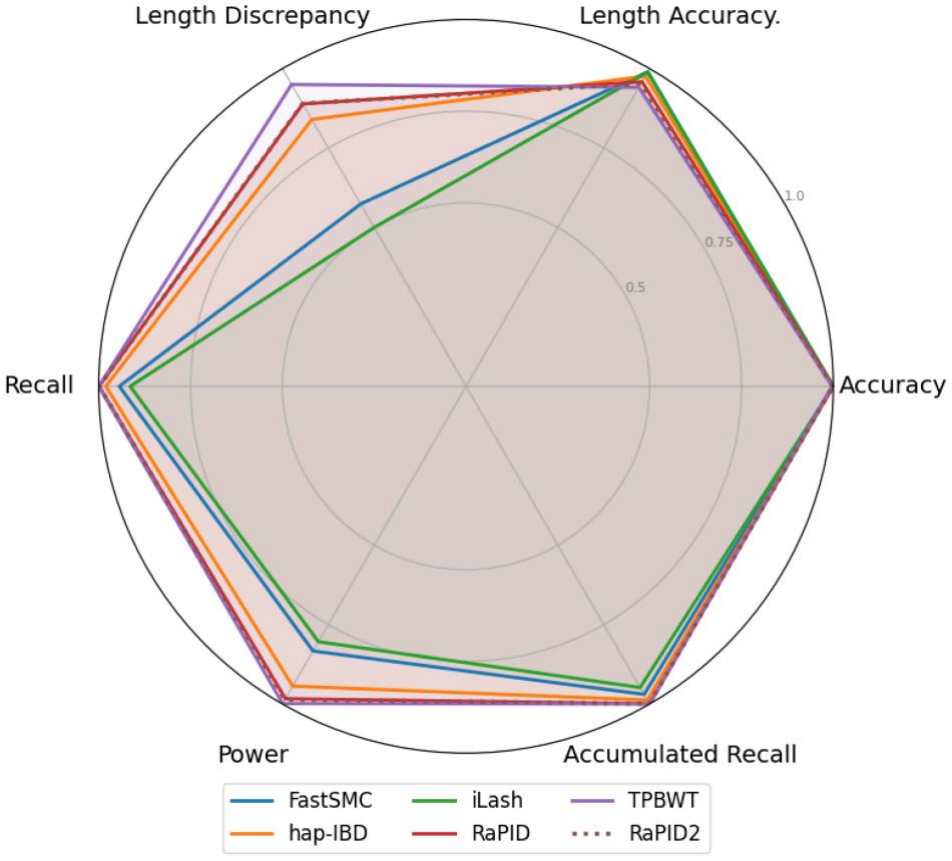
Power and accuracy results at a 5 cM length threshold from the IBD segment detection tool benchmarking project, evaluated on msprime OOA model EUR array data (Kelleher *et al*. 2016) with a simulated genotyping error rate of 0.1%. The *Length Discrepancy* is normalized from genetic distance and quantifies the agreement between detected and true IBD segment lengths; all other values are reported as percentages. Raw data for this figure is provided in Table 2, available as supplementary data at *Bioinformatics Advances* online. The parameters and command lines used for each tool are provided in Table 3, available as supplementary data at *Bioinformatics Advances* online.

The six metrics used in Figs 3 and 4 follow the same definitions from the open-source IBD detection benchmarking project: *Accuracy* is the fraction of reported segments overlapping a true segment by at least 50% of their length; *Length discrepancy* captures the absolute difference between them in cM; *Length accuracy* measures the ratio between reported and true segment lengths; *Recall* is the fraction of true segments detected; *Power* counts unique true segments hit by at least one call; *Accumulated recall* integrates recall across length bins; and *Length accuracy* measures the ratio between reported and true segment lengths. False positive rate corresponds to 1−Accuracy.

While our focus is on confirming the fidelity of RaPID2 relative to RaPID, the benchmarking setup includes additional tools such as hap-IBD, iLASH (Shemirani *et al.* 2021), FastSMC (Nait Saada *et al.* 2020) and TPBWT (Freyman *et al.* 2021) as part of the original evaluation framework. Figures 3 and 4 show minor numerical differences between RaPID and RaPID2, which are entirely attributable to stochastic variation inherent in randomized methods. Critically, all power and accuracy metrics confirm that RaPID2 replicates the behavior of RaPID with high fidelity, supporting the conclusion that any observed differences in performance are implementation-neutral and that RaPID2 inherits the same inferential properties while achieving major computational gains.

### 3.2 Benchmarking environment

To demonstrate the computational performance of RaPID2, we collected performance measurements using the HPC mode of RaPID2, its predecessor RaPID, as well as the widely used and well-established hap-IBD as a reference. The runtime and memory consumption benchmark uses UK Biobank chromosome 20, which contains 974 818 haplotypes and 17 197 markers at the time of the experiment. All benchmarking experiments were conducted on a high-performance compute node equipped with 128 logical cores and 1 TB of RAM (Intel Xeon Gold 6338 N @ 2.20 GHz). Performance was evaluated using four primary metrics: (i) peak memory, defined as the highest resident memory usage recorded during execution; (ii) CPU time, which represents the total sum of processing time across all threads; (iii) wall-clock time, measuring end-to-end runtime from process start to completion; and (iv) CPU utilization, computed as total CPU time divided by wall-clock time and the number of logical cores (i.e. parallel efficiency).

Although peak memory usage provides a practical indication of demand, it should not be interpreted as a strict lower bound for successful execution. Most modern operating systems and runtimes, including those used in high-performance computing-adopt non-immediate memory release strategies such as lazy garbage collection, deferred heap reclamation, and memory pooling. In these environments, memory no longer in active use may remain allocated to the process for extended periods, either to reduce fragmentation or to optimize future allocations. As a result, runtime monitoring tools may report elevated memory usage that does not reflect sustained or critical resource pressure. This discrepancy is especially pronounced in Memory-Retentive workloads like RaPID2, where large in-memory buffers are frequently reused. In such cases, temporarily inflated peak memory readings may occur even when actual memory pressure remains stable (Kim *et al.* 2024).

While hap-IBD employs a fundamentally different algorithmic approach with distinct power and accuracy characteristics, it remains a widely adopted method and an influential benchmark in IBD detection. Despite exhibiting lower detection power under certain parameter settings, hap-IBD is included here to provide reference points due to its prominence in the field and widespread usage in recent population-scale studies.

For RaPID, execution at the 2 cM threshold was not performed on the primary HPC server due to the excessive runtime and disk IO demands involved. Running such a long-duration, file-intensive process on a shared high-performance node is inefficient and incompatible with best practices for computing resource allocation. Instead, this experiment was conducted on a dedicated workstation with an Intel(R) Core(TM) i7-3770 CPU @ 3.40 GHz, featuring 8 vCPUs and 32 GB of RAM. For all experiments labeled as RaPID, we used the latest available version, RaPID v1.7. To ensure consistent cross-method comparisons, we adjusted the resulting runtime by a CPU clock-normalized factor to estimate the equivalent performance if RaPID had been run on the 128-core Xeon Gold 6338N HPC node. This adjusted runtime is used only for reporting benchmark ratios and cloud price estimates, and is clearly denoted where applicable.

All experiments were executed using the Memory-Retentive Mode, which retains all necessary PBWT structures and random projections in memory throughout execution. For each input configuration, we empirically determined the smallest number of partitions that allowed the complete analysis to execute without exceeding the 1 TB physical memory limit of the benchmarking node. This partition count was selected to prioritize runtime efficiency while remaining within feasible memory constraints. It should be noted that while increasing the number of partitions generally reduces memory usage per partition, it also incurs additional overhead due to repeated computation across partitions, thereby increasing total wall-clock time. However, this trade-off between partition granularity and runtime is not strictly linear. Smaller partition sizes reduce peak memory stress and may improve garbage collection and cache locality, leading to sublinear increases in runtime. Moreover, server-level behaviors such as memory paging, thread scheduling, and IO buffering can introduce variability across systems. As a result, the relationship between partition count and runtime is highly dependent on server architecture, operating system configuration, and memory management policies. In addition, the same hardware platform may exhibit distinct memory and CPU utilization profiles for different genomic datasets, due to underlying panel characteristics such as sample size, haplotype diversity, and local recombination rates.

### 3.3 Benchmark results

**Figure 5:**
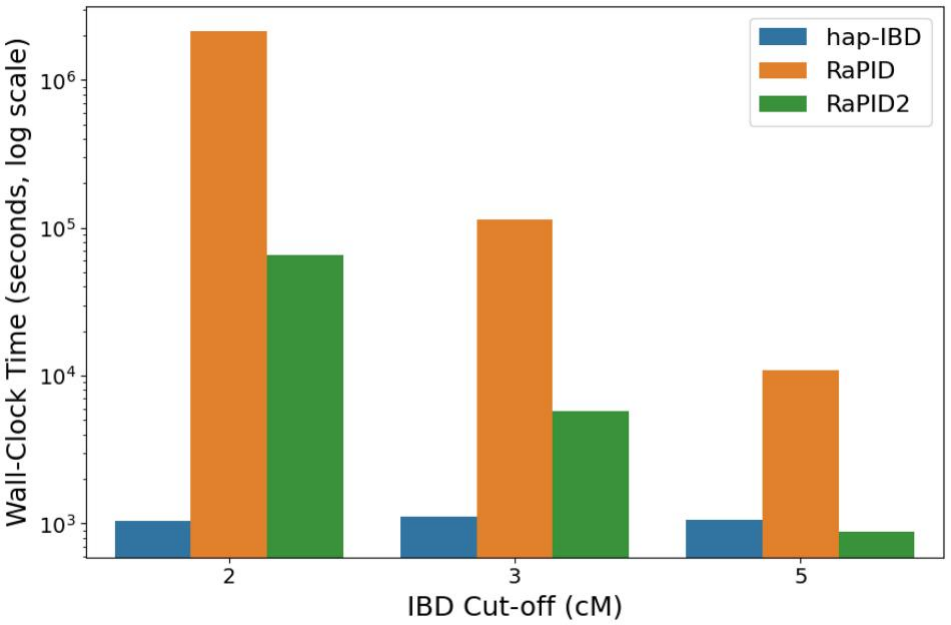
Wall-clock time (log scale) for three IBD detection tools across different IBD cut-off values. Each group of bars compares hap-IBD, RaPID, and RaPID2 on UK Biobank chromosome 20.

Figure 5 illustrates the wall-clock time performance of hap-IBD, RaPID, and RaPID2 across varying IBD detection thresholds. Table 2 presents detailed benchmark results, including peak memory usage, CPU time, wall-clock time, and observed CPU utilization.

**Table 2:** Benchmarking results for hap-IBD, RaPID, and RaPID2 (HPC Mode) on UK Biobank chromosome 20.^a^

Method	IBD cut-off (cM)	Peak memory (GB)	CPU time (s)	Wall time (s)	CPU util. (%)	Speedup (×)
hap-IBD	2	257	70 298	1045	56%	—
RaPID	2	7.25	2 131 098	2 131 098	N/A	—
RaPID2 (*p* = 100)	2	822	2 746 397	65 513	35%	32.53
hap-IBD	3	162	31 919	1123	24%	—
RaPID	3	2.83	114 334	114 334	N/A	—
RaPID2 (*p* = 10)	3	759	434 216	5720	63%	19.99
hap-IBD	5	127	31 919	1068	25%	—
RaPID	5	2.73	10 868	10 868	N/A	—
RaPID2 (*p* = 4)	5	819	76 739	875	73%	12.42

a Reported values include peak memory usage (GB), total CPU time (seconds), wall-clock time (seconds), and CPU utilization as a percentage of 128 logical cores. The speedup column denotes how many times faster RaPID2 is compared to RaPID under identical output settings. *p* donates the number of partitions used for RaPID2 in memory-retentive (HPC) mode to balance runtime and memory load.

At the 5 cM threshold, RaPID2 demonstrates a substantial runtime advantage, completing the task in 875 seconds, 12.4× faster than RaPID, while maintaining a high CPU utilization of 73%. The memory footprint remains under 1 TB even at minimal partitioning (*p* = 4), showcasing the efficiency of the Memory-Retentive Mode on well-structured, coarse-resolution workloads.

As the threshold is lowered to 3 cM, computational demand increases for all tools. RaPID2 maintains a 19.99× improvement over RaPID while operating with 759 GB peak memory at 10 partitions. CPU utilization remains high (63%), indicating efficient core usage and balanced memory access even at higher resolution.

At the most challenging 2 cM setting, RaPID2 completes this task in approximately 18.2 hours with 100 partitions, using 822 GB of memory and delivering a 32.5× speedup over RaPID. While CPU utilization at this setting is lower (35%), the memory-bound nature of the computation remains evident and consistent with profiling results.

hap-IBD exhibits substantially lower memory consumption, its runtime performance is excellent at both the 2 cM and 3 cM thresholds, completing in under 20 minutes for each. While RaPID2 does not surpass hap-IBD in raw runtime at these settings, its performance remains within a practically reasonable range: for example, RaPID2 completes the 2 cM benchmark in under one day, compared to hap-IBD’s 17 min. This gap is acceptable given that RaPID2 is a high-power method based on RaPID, capable of matching its output exactly, whereas hap-IBD follows a different algorithmic approach and has been reported to yield lower detection power in certain scenarios. More importantly, RaPID2 delivers an enormous improvement over RaPID itself, reducing runtime from nearly one month to under 24 hours. This makes high-resolution IBD detection on large cohorts far more accessible, without sacrificing power and accuracy.

Overall, these benchmarks demonstrate that RaPID2 preserves the detection power and output structure of RaPID while achieving 12 to 32× runtime improvements across all tested thresholds. These gains are obtained through a memory-aware architecture that eliminates disk usage, minimizes redundant computation, and enables efficient partition scaling across a range of execution environments.

### 3.4 Internal partition analysis for RaPID2

We further evaluated RaPID2 under different memory configurations by varying the number of partitions (*p*) used in its Memory-Retentive execution mode. Table 3 shows that increasing the number of partitions results in reduced peak memory usage but generally longer runtime. While runtime scales with *p* in a predictable trend, the observed memory peaks are less reliable due to the server’s lazy memory reclamation behavior, which may defer freeing previously used memory and inflate peak estimates. Nevertheless, the trend supports the intuitive trade-off: more partitions lower memory demand but introduce greater IO coordination and repeated computation, thereby increasing wall-clock time.

**Table 3:** RaPID2 performance on UK Biobank chromosome 20 (2 cM cut-off) under varying partition counts.^a^

Partitions	Peak memory (GB)	Wall time (s)
100	822	65 513
200	693	91 447
400	634	152 447

a Peak memory usage (GB) and wall-clock time (seconds) are reported. Memory values reflect maximum observed usage and may not represent the strict minimum required allocation.

### 3.5 Cost estimation and hardware efficiency

To assess the cloud-based cost of executing RaPID and RaPID2, we simulate their runtimes on AWS EC2 instances with comparable architecture to the nodes used in our institutional high-performance computing (HPC) cluster. Due to UK Biobank data access restrictions, all experiments were conducted locally, and the EC2 estimates presented here are intended solely for comparative cost modeling, not as direct performance benchmarks. We selected instances from the r7iz family, which features Intel Sapphire Rapids CPUs, to ensure consistency across both versions. The local reference runtime and estimated cloud execution costs for both methods are summarized in Table 4.

**Table 4:** Estimated EC2 runtime and cost for RaPID and RaPID2 on UK Biobank chromosome 20 with 2 cM cut-off.^a^

Version	Instance	vCPU	Memory	Storage	Hourly rate	Reference hours	Estimated cost
RaPID	r7iz.xlarge	4	32 GB	1 TB EBS	$0.372 + $0.114	591.97	$287.56
RaPID	r7iz.xlarge	4	32 GB	2 TB EBS	$0.372 + $0.228	591.97	$354.92
RaPID2	r7iz.12xlarge	48	768 GB	500 GB EBS	$4.128 + $0.057	18.20	$76.17
RaPID2	r7iz.16xlarge	64	1024 GB	500 GB EBS	$5.504 + $0.057	18.20	$101.21

a Reference hours are wall-clock runtimes observed on local server and used for cost modeling. Hourly rates include both compute and temporary storage costs. Pricing is based on on-demand EC2 instance rates at the time of writing Amazon Web Services (2025).

For RaPID, we assume execution on an r7iz.xlarge instance (4 vCPUs, 32 GB RAM) with an hourly on-demand rate of $0.372. This configuration is sufficient for the largely sequential workload of RaPID. However, due to the generation of large intermediate files during long match detection and merging, persistent disk storage is required. We consider two EBS configurations: a 1 TB and a 2 TB volume, priced at $0.08/GB-month, yielding additional storage costs of approximately $67.35 and $134.71 respectively for a 591.97-hour run.

Although EBS volumes are flexible and persistent, they are also network-attached and introduce latency. RaPID involves intensive read and write activity, which makes EBS a poor fit in practice. To address this, one could utilize onboard SSD (instance store) volumes, which offer much lower latency and higher throughput. These SSDs are directly attached to the host and are included at no additional cost on certain EC2 instances. However, r7iz.xlarge does not provide instance store volumes. The smallest available configuration that includes onboard SSD is r7iz.12xlarge (48 vCPUs, 768 GB RAM), priced at $4.128/hour. Running RaPID on this instance for 591.97 hours would result in a cost of $2443.65.

To summarize, while our primary cost estimates use r7iz.xlarge with EBS to provide a lower-bound dollar estimate for RaPID, we caution that this comes at the expense of performance. EBS-backed runs may take significantly longer than the assumed 592 hours. In contrast, onboard SSD usage would yield much faster IO but requires more expensive compute instances. This trade-off between cost, runtime, and hardware availability should guide configuration decisions for large-scale deployments.

For RaPID2, we analyze two configurations: r7iz.12xlarge (48 vCPUs, 768 GB RAM, $4.128/hour) and r7iz.16xlarge (64 vCPUs, 1024 GB RAM, $5.504/hour), then include 500 GB disk storage to each configuration. These instances reflect the memory-centric design of RaPID2, which performs all computation in memory without generating temporary files. Runtime profiling shows that CPU utilization remains below 50%, confirming that RaPID2 is memory-bound. To validate this behavior, we also conducted tests on our departmental HPC server and observed no significant runtime improvement when increasing from 48 to 64 or 128 cores. Based on this behavior, we use a shared runtime estimate of 18.20 h for both cloud configurations when modeling cost. This suggests that RaPID2 does not scale proportionally with additional cores beyond a certain point, likely due to memory bandwidth and internal workload structure. Consequently, we consider r7iz.12xlarge a cost-efficient and performance-equivalent choice for cloud deployment. Nonetheless, selecting r7iz.16xlarge remains a reasonable and computationally robust configuration in settings where higher memory capacity or simplified workload distribution is desired.

Estimated cost savings. Compared to the lowest-cost RaPID scenario ($287.56), RaPID v reduces total cost by: 73.9% using r7iz.12xlarge ($75.13); 65.2% using r7iz.16xlarge ($100.17).

If we instead consider the IO-efficient but more expensive RaPID run on r7iz.12xlarge with onboard SSD ($2443.65), the savings from RaPID2 range from 95.9% to 96.9%, highlighting the substantial benefit of memory-aware, disk-free design.

In addition to reducing cost, RaPID2 drastically improves turnaround time. While RaPID requires nearly 25 days to complete the 2 cM scan on chromosome 20, RaPID2 completes the same task in under 19 hours, a speed-up of over 32×. This acceleration enables practical high-resolution IBD detection at scale, even when operating under tight computational budgets or shared environments. For large-cohort studies or iterative analysis pipelines, this improvement in delivery time is often as impactful as the reduction in monetary cost.

## 4 Conclusion and discussion

The development of RaPID2 represents a substantial advancement in IBD detection for biobank-scale genomic data. By integrating algorithmic innovations with systems-level redesign, RaPID2 overcomes key bottlenecks of RaPID while preserving its statistical strengths. Our benchmarking confirms that RaPID2 achieves significant runtime reductions up to 32-fold faster than RaPID at 2 cM, while maintaining comparable power to established tools such as hap-IBD.

RaPID2 introduces two execution modes: a Memory-Stable mode compatible with general purpose machines and an in-memory Memory-Retentive (HPC) mode optimized for large-memory compute nodes. This flexibility allows the tool to be deployed across a range of computing contexts, from sequential academic servers to parallelized cloud environments. Our results demonstrate that both modes produce identical outputs, differing only in runtime and memory characteristics. The Memory-Stable mode is fault-tolerant and conservative in memory use, while the HPC mode enables high-throughput inference at minimal wall-clock time.

While RaPID2 achieves substantial cost and time savings under HPC mode execution, our cost analysis emphasizes that such performance gains depend on memory availability and compute pricing. The estimated EC2 deployment cost for RaPID2 using 768 GB memory was over 70% lower than for RaPID on comparable data, and up to 96% lower when considering realistic IO constraints. In addition to financial savings, RaPID2 reduces delivery time from nearly a month to under a day, offering immediate practical advantages for time-sensitive projects.

Unlike RaPID’s file-intensive and sequential design, RaPID2 adopts a fully in-memory architecture, supported by cache-aware match processing, region-aware parallelization, and lock-free accumulation strategies. These updates eliminate IO as a limiting factor and reduce CPU idling, resulting in high hardware utilization. By preserving match locality and respecting memory hierarchy, RaPID2 adapts naturally to modern multi-core systems.

Despite structural changes, RaPID2 retains the statistical principles of RaPID. The random projection layer, now generalized to support dynamic, fixed, or hybrid windowing, ensures adaptability across panels of varying SNP density and recombination rates. To validate fidelity, we configured RaPID2 to produce output identical to RaPID under benchmark conditions, affirming that our improvements are systems-level rather than statistical.

RaPID2 is designed to maximize throughput, not minimal memory usage. While the partitioned approach can mitigate peak memory, extremely constrained environments may still present challenges. Additionally, although we have shown that RaPID2 performs competitively with hap-IBD in terms of runtime and power, hap-IBD uses a fundamentally different algorithmic framework and may outperform RaPID2 in specific short cut-off scenarios. We do not claim universal superiority, but rather emphasize that RaPID2 offers a complementary approach with distinct architectural advantages.

Moreover, our current benchmark uses fixed random projections with uniform windowing, but the framework is amenable to data-driven adaptations. For example, genomic regions with known structural complexity or local ancestry variation could benefit from dynamic adjustments to window size or projection density. While not explored in this study, such strategies could further improve power and efficiency.

RaPID2 offers a robust, flexible, and high-performance framework for IBD detection that is compatible with both traditional and modern computing infrastructure. It bridges algorithmic accuracy and engineering efficiency, enabling scalable analysis across datasets that would overwhelm legacy tools. As genomic resources continue to expand in size and complexity, tools like RaPID2 will be essential in meeting the dual demands of statistical precision and computational scalability.

## Supplementary Material

vbag078_Supplementary_Data

## Data Availability

RaPID2 is implemented in C#. Its source code and software package are available at https://github.com/ucfcbb/RaPID2.

## References

[vbag078-B1] All of Us Research Program Investigators. The “All of Us” research program. N Engl J Med 2019;381:668–76. 31412182 10.1056/NEJMsr1809937 PMC8291101

[vbag078-B2] Amazon Web Services. Amazon EC2 On-Demand Pricing. 2025. https://aws.amazon.com/ec2/pricing/on-demand/ (July 2025, date last accessed).

[vbag078-B3] Blelloch GE. Scans as Primitive Parallel Operations. IEEE Trans Comput 1989;38:1526–38.

[vbag078-B4] Bokhari SH. A general Technique for Partitioning Graphs. IEEE Trans Software Eng 1978;SE-4:637–48.

[vbag078-B5] Browning BL , Browning SR. A fast, powerful method for detecting identity by descent. Am J Hum Genet 2011;88:173–82. 21310274 10.1016/j.ajhg.2011.01.010 PMC3035716

[vbag078-B6] Dean J , Ghemawat S. Mapreduce: simplified data processing on large clusters. Commun ACM 2008;51:107–13.

[vbag078-B7] Devine KD , Boman E, Karypis G. Partitioning and Load Balancing for Emerging Parallel Applications and Architectures. In: Recent Advances in Parallel Virtual Machine and Message Passing Interface, Philadelphia, PA, 2006, 99-126.

[vbag078-B8] Ding N , Maris P, Nam HA et al Evaluating the potential of disaggregated memory systems for HPC applications. Concurr Comput Pract Exp 2024;36:e8147.

[vbag078-B9] Durbin R. Efficient haplotype matching and storage using the positional Burrows–Wheeler transform (PBWT). Bioinformatics 2014;30:1266–72. 24413527 10.1093/bioinformatics/btu014 PMC3998136

[vbag078-B10] Freyman WA , McManus KF, Shringarpure SS et al; 23 and Me Research Team. Fast and Robust Identity-by-Descent Inference with the Templated Positional Burrows–Wheeler Transform. Mol Biol Evol 2021;38:2131–51. 33355662 10.1093/molbev/msaa328 PMC8097300

[vbag078-B11] Gusev A , Lowe JK, Stoffel M et al Whole population, genome-wide mapping of hidden relatedness. Genome Res 2009;19:318–26. 18971310 10.1101/gr.081398.108 PMC2652213

[vbag078-B12] Gutenkunst RN , Hernandez RD, Williamson SH et al Inferring the Joint Demographic History of Multiple Populations from Multidimensional SNP Frequency Data. PLoS Genet 2009;5:e1000695. 19851460 10.1371/journal.pgen.1000695 PMC2760211

[vbag078-B13] Han E , Carbonetto P, Curtis RE et al Clustering of 770,000 genomes reveals post-colonial population structure of North America. Nat Commun 2017;8:14238. 28169989 10.1038/ncomms14238 PMC5309710

[vbag078-B14] Henn BM , Hon L, Macpherson JM et al Cryptic Distant Relatives Are Common in Both Isolated and Cosmopolitan Genetic Samples. PLoS One 2012;7:e34267. 22509285 10.1371/journal.pone.0034267 PMC3317976

[vbag078-B15] Herlihy M , Liu Z. Well-structured futures and cache locality. ACM SIGPLAN Notices 2024;49:155–166.

[vbag078-B16] Herlihy M , Shavit N. The Art of Multiprocessor Programming. San Francisco, CA, USA: Morgan Kaufmann, 2008.

[vbag078-B17] Kelleher J , Etheridge AM, McVean G et al Efficient Coalescent Simulation and Genealogical Analysis for Large Sample Sizes. PLoS Comput Biol 2016;12:e1004842. 27145223 10.1371/journal.pcbi.1004842 PMC4856371

[vbag078-B18] Kim D , Brown T, Singh A. Are Your Epochs Too Epic? Batch Free Can Be Harmful. In: Proceedings of the 29th ACM SIGPLAN Annual Symposium on Principles and Practice of Parallel Programming, Edinburgh, UK. ACM, New York, NY, USA, 2024, 30–41.

[vbag078-B24] Kogge PM , Stone HS. A Parallel Algorithm for the Efficient Solution of a General Class of Recurrence Equations. IEEE Trans Comput 1973;C-22:786–793.

[vbag078-B19] Mansi M , Swift M. Characterizing Physical Memory Fragmentation. arXiv preprint arXiv*:*2401.03523, 2024.

[vbag078-B20] Merrill D , Garland M. Single-Pass Parallel Prefix Scan with Decoupled Look-Back. Technical Report. NVIDIA Corporation, Santa Clara, CA, USA, 2016.

[vbag078-B21] Musser DR. Introspective Sorting and Selection Algorithms. Softw Pract Exp 1997, 983–93.

[vbag078-B22] Nait Saada J , Kalantzis G, Shyr D et al Identity-by-descent detection across 487,409 British samples reveals fine scale population structure and ultra-rare variant associations. Nat Commun 2020;11:6130–15. 33257650 10.1038/s41467-020-19588-x PMC7704644

[vbag078-B23] Naseri A , Liu X, Tang K et al RaPID: ultra-fast, powerful, and accurate detection of segments identical by descent (IBD) in biobank-scale cohorts. Genome Biol 2019;20:143–15. 31345249 10.1186/s13059-019-1754-8 PMC6659282

[vbag078-B25] Pagter J , Rauhe T. Optimal time-space trade-offs for sorting. In: *Proceedings 39th Annual Symposium on Foundations of Computer Science*. Palo Alto, CA, USA. IEEE, Piscataway, NJ,USA, 1998, 264–8.

[vbag078-B26] Palamara PF , Lencz T, Darvasi A et al Length Distributions of Identity by Descent Reveal Fine-Scale Demographic History. Am J Hum Genet 2012;91:809–22. 23103233 10.1016/j.ajhg.2012.08.030 PMC3487132

[vbag078-B27] Shemirani R , Belbin GM, Avery CL et al Rapid detection of identity-by-descent tracts for mega-scale datasets. Nat Commun 2021;12:3546. 34112768 10.1038/s41467-021-22910-w PMC8192555

[vbag078-B28] Stoeklé H-C , Mamzer-Bruneel M-F, Vogt G et al 23andMe: a new two-sided data-banking market model. BMC Med Ethics 2016;17:19–1. 27059184 10.1186/s12910-016-0101-9 PMC4826522

[vbag078-B29] Sudlow C , Gallacher J, Allen N et al UK Biobank: An Open Access Resource for Identifying the Causes of a Wide Range of Complex Diseases of Middle and Old Age. PLoS Med 2015;12:e1001779. 25826379 10.1371/journal.pmed.1001779 PMC4380465

[vbag078-B30] Sun X-H , Ni LM. Scalable Problems and Memory-Bounded Speedup. J Parallel Distrib Comput 1993;19:27–37.

[vbag078-B31] Tang K , Naseri A, Wei Y et al Open-source benchmarking of IBD segment detection methods for biobank-scale cohorts. Gigascience 2022;11 giac111. 36472573 10.1093/gigascience/giac111 PMC9724555

[vbag078-B32] Tang K , Sanaullah A, Zhi D et al Haplotype-based Parallel PBWT for Biobank Scale Data. In: *Proceedings of the 2025 IEEE International Conference on Computational Advances in Bio and Chemical Systems (ICCABC)*. Atlanta, GA, USA. IEEE, Piscataway, NJ, USA, 2025, 130–43. 10.1007/978-3-032-02489-3_10 PMC13178698 42147361

[vbag078-B33] Vaithianathan M. Memory Hierarchy Optimization in High Performance Computing. J Comput Archit High Perform Comput 2025;34:1–15.

[vbag078-B34] Xuan P , Luo F, Ge R et al Dynims: a dynamic memory controller for in-memory storage on HPC systems. arXiv preprint arXiv:1609.09294, 2016.

[vbag078-B35] Zaharia M , Chowdhury M, Franklin MJ et al Spark: Cluster Computing with Working Sets. In: *Proceedings of the 2nd USENIX Conference on Hot Topics in Cloud Computing*, Boston, MA, USA: USENIX Association, Berkeley, CA, USA, 2010, 10.

[vbag078-B36] Zhou Y , Browning SR, Browning BL et al A Fast and Simple Method for Detecting Identity-by-Descent Segments in Large-Scale Data. Am J Hum Genet 2020;106:426–37. 32169169 10.1016/j.ajhg.2020.02.010 PMC7118582

