# Supplementary material for: RaPID2: a parallel scalable framework for identity-by-descent segment detection via parallel PBWT
Source: Bioinform Adv. 2026 Mar 17;6(1):vbag078. doi: 10.1093/bioadv/vbag078 (PMC13049448; doi:10.1093/bioadv/vbag078)
Supplement: vbag078_Supplementary_Data [file vbag078_supplementary_data.pdf]

Supplementary Materials for "RaPID2: A Parallel Scalable Framework for Identity-by-Descent Segment Detection via Parallel PBWT"

Kecong Tang[1], Ardalan Naseri[2], Degui Zhi[2] and Shaojie Zhang[1]

[1]Department of Computer Science, University of Central Florida, FL, 32816, USA
[2]McWilliams School of Biomedical Informatics, The University of Texas Health Science Center at Houston, TX, 77030, USA

Supplementary Tables

Table S1: Benchmarking metrics used to generate the radar plot (Figure 3), derived from the IBD segment detection tool benchmarking project using a 2 cM length threshold on msprime OOA model EUR array data [28] with a simulated genotyping error rate of 0.1%. The length discrepancy is measured by cM; other measures are based on percentage.

**Tool**	**Accuracy**	**Length Acc.**	**Length Disc.**	**Recall**	**Power**	**Acc. Recall**
FastSMC	0.9993	0.9768	0.8606	0.4353	0.3902	0.4353
hap-IBD	0.9975	0.9343	0.3954	0.7731	0.7574	0.7731
iLash	0.9984	0.9607	0.8870	0.6427	0.6188	0.6427
RaPID	0.9898	0.8882	0.4224	0.9711	0.9665	0.9711
TPBWT	0.9797	0.8491	0.4918	0.9704	0.9689	0.9704
RaPID2	0.9879	0.8752	0.4447	0.9761	0.9720	0.9761

Table S2: Benchmarking metrics used to generate the radar plot (Figure 4), derived from the IBD segment detection tool benchmarking project using a 5 cM length threshold on msprime OOA model EUR array data [28] with a simulated genotyping error rate of 0.1%. The length discrepancy is measured by cM; other measures are based on percentage.

Tool	Accuracy	Length Acc.	Length Disc.	Recall	Power	Acc. Recall
FastSMC	0.9998	0.9901	2.4267	0.9440	0.8332	0.9698
hap-IBD	0.9996	0.9764	1.0297	0.9808	0.9438	0.9875
iLash	0.9997	0.9887	2.8148	0.9145	0.8041	0.9482
RaPID	0.9989	0.9589	0.7718	0.9989	0.9834	0.9994
TPBWT	0.9965	0.9410	0.4508	0.9999	0.9986	0.9999
RaPID2	0.9989	0.9535	0.7666	0.9985	0.9863	0.9990

Table S3: Parameters and commands used for each tool to generate the radar plot (Figure 3 and 4), derived from the IBD segment detection tool benchmarking project.

Tool	Command / Parameters
**FastSMC**	`--min_m 2`
	`bcftools convert ge.seq.e0.000125.vcf --hapsample ge.seq.e0.000125`
	`./FastSMC_exe --inFileRoot ge.seq.e0.000125 \`
	`--outFileRoot F_ge.seq.e0.000125_2 \`
	`--decodingQuantFile decodingQuantities.gz \`
	`--mode array --min_m 2 --segmentLength --hashing \`
	`--perPairPosteriorMeans --perPairMAP --noConditionalAgeEstimates`
**hap-IBD**	`min-output=2`
	`java -Xmx50g -jar hap-ibd.jar gt=ge.seq.e0.000125.vcf \`
	`out=H_ge.seq.e0.000125_2cM map=ge.seq.e0.0005.vcf.pMap min-output=2`
**iLash**	`ped ge.seq.e0.000125.ped`
	`map ge.seq.e0.000125.map`
	`output I_ge.seq.e0.000125_2.match`
	`slice_size 350; step_size 350; perm_count 20;`
	`shingle_size 15; shingle_overlap 0; bucket_count 5;`
	`max_thread 20; match_threshold 0.99; interest_threshold 0.70;`
	`max_error 0; min_length 2; auto_slice 1; cm_overlap 1;`
	`minhash_threshold 55`
	`./IBD iLa_ge.seq.e0.000125_2.config`
**RaPID**	`w=30 (30 for sequencing, 3 for array)`
	`r=10; s=2; d=2`
	`./RaPID_v.1.7 -i ge.seq.e0.000125.vcf.gz -o ge.seq.e0.000125.vcf_2w_30 \`
	`-w 30 -r 10 -s 2 -d 2 -g ge.seq.e0.0005.vcf.rMap`
**TPBWT**	`use_phase_correction=False; L_f=2`
	`runTPBWT.py:`
	`import phasedibd as ibd`
	`haplotypes = ibd.VcfHaplotypeAlignment(path+sys.argv[2], path+sys.argv[3])`
	`tpbwt = ibd.TPBWTAnalysis()`
	`ibd_results = tpbwt.compute_ibd(haplotypes, L_f=float(sys.argv[1]),`
	`use_phase_correction=False, segments_out_path=`
	`path+"T_"+sys.argv[2]+"_"+sys.argv[1]+"cM.csv")`
	`python3 runTPBWT.py 2 ge.seq.e0.000125.vcf ge.seq.e0.0005.vcf.pMap`
